# Pancreatic Cancer Biopsy Modalities: Comparing Insurance Status, Length of Stay, and Hospital Complications Based on Percutaneous, Endoscopic, and Surgical Biopsy Methods

**DOI:** 10.7759/cureus.39660

**Published:** 2023-05-29

**Authors:** Anmol Mittal, Alexander Le, Aaron Kahlam, Syed F Haider, Vishnu Prasath, Ayham Khrais, Ravi Chokshi

**Affiliations:** 1 Department of Medicine, Rutgers University New Jersey Medical School, Newark, USA; 2 Department of General Surgery, Rutgers University New Jersey Medical School, Newark, USA; 3 Department of Surgical Oncology, Rutgers University New Jersey Medical School, Newark, USA

**Keywords:** hospital complication, endoscopic, pancreatic cancer, pancreatic biopsy, pancreas lesion

## Abstract

Background: Pancreatic cancer is diagnosed histologically through percutaneous biopsy (PB), endoscopic biopsy (EB), or surgical biopsy (SB). Factors and outcomes associated with method type are not clearly understood. We aimed to evaluate the relationship between insurance status, length of hospital stay (LOS), complications, and different pancreatic biopsy modalities.

Study: The 2001-2013 database from the National (Nationwide) Inpatient Sample (NIS) was queried for those with pancreatic cancer who underwent biopsies using International Classification of Diseases, Ninth Revision (ICD-9) codes. Data regarding insurance status, hospital stay, demographics, and complications were analyzed using chi-square and multivariate analysis with α < 0.001.

Results: A total of 824,162 patients with pancreatic cancer were identified. Uninsured and Medicaid patients were more likely to get PB compared to SB. Patients were more likely to have acute renal failure (ARF) with an EB compared to SB. Patients were more likely to have a urinary tract infection (UTI) with EB or PB compared to SB. All biopsy types were less likely to have pneumonia; pancreatitis was more prevalent in EB compared to PB and SB.

Conclusions: Uninsured and Medicaid patients were most likely to have a PB compared to EB despite unclear indications which may represent an underlying discrepancy in healthcare utilization. EB patients had the shortest LOS while SB patients stayed three more days; those who underwent a combination of biopsies had the greatest LOS. Patients with EB were more likely to develop ARF, UTI, and pancreatitis than SB, possibly attributed to the advanced nature of endoscopic ultrasound. It is important to establish appropriate algorithm contributors to guide decision-making.

## Introduction

Pancreatic ductal adenocarcinoma is one of the leading causes of cancer death worldwide. In the United States, there were an estimated 57,000 new cases in 2020. It is a highly fatal malignancy with a five-year surgical rate of 10% in the United States. The majority of patients present in late stages, with either metastatic or unresectable disease. Additionally, prognosis remains poor for a small subset of patients who are diagnosed with localized and resectable disease with only 20% surviving over a five-year period [1]. Diagnostic evaluation of a patient with pancreatic masses starts with serological evaluation and abdominal imaging, such as computed tomography (CT), and magnetic resonance cholangiopancreatography (MRCP). Additionally, CT imaging can facilitate the staging of the disease by identifying the presence or absence of metastatic disease [2-4].

While imaging helps identify the presence of pancreatic masses, histological analysis is required to establish a diagnosis of pancreatic cancer and to differentiate adenocarcinoma from other benign lesions. However, pancreatic biopsy is an invasive diagnostic method and carries significant risk. Other indications for a pancreatic biopsy include advanced pancreatic neoplasia requiring neoadjuvant therapy, areas suspected of containing lesions for which treatment is primarily nonsurgical, and high-surgical risk patients [5].

In cases when ultrasound or CT allows for large pancreatic masses to be seen, the typical next step that is currently in place is using extraction methods with surgery and concurrently performing a surgical biopsy (SB). However, when there is insufficient evidence of pancreatic cancer, there are two other options to obtain a tissue sample: CT or ultrasound-guided percutaneous core needle biopsy or endoscopic biopsy (EB). Typically, biopsies at the tail of the pancreas are approached percutaneously or surgically, while small to medium-sized lesions at the body and head are approached endoscopically [6,7].

Other than tumor size and location, other factors impacting the decision to pursue one of these three modalities remain unclear for pancreatic biopsy. Additionally, there have been limited studies comparing outcomes between the three methods [8,9]. The objectives of this study are to identify differences in biopsy methods by insurance status and to analyze differences in length of hospital stay (LOS) and hospital complication rates in patients with pancreatic cancer.

This article was previously presented as a meeting abstract at the 2020 American College of Gastroenterology (ACG) Annual Scientific Meeting on October 23, 2020 [10].

## Materials and methods

Data source/study population

We used the National (Nationwide) Inpatient Sample (NIS) 2001-2013 database to retrospectively identify patients with a primary diagnosis of pancreatic cancer. This database was queried for patients with pancreatic cancer using International Classification of Diseases, Ninth Revision (ICD-9) codes 157.0-157.9. Percutaneous biopsy (PB), EB, and SB were also identified with ICD-9 procedure codes 52.11, 52.13 + 52.14, and 52.12, respectively. This was a retrospective study that did not involve active recruitment or enrollment of patients. As such, informed consent was not required by the institutional review boards.

Study variables/outcome

Patients with pancreatic cancer were categorized into four groups based on the type of biopsy they had: SB, PB, EB, or a combination of the three. After patients were stratified, they were then studied based on the following: (1) Insurance status (i.e. Medicaid vs. Medicare vs. uninsured), (2) Average LOS in the hospital, (3) Complications during hospital stay (myocardial infarction (MI), pancreatitis, pneumonia, urinary tract infection (UTI), and acute renal failure (ARF)). Patients were also studied based on age, race, gender, and insurance status.

Patient characteristics and demographics 

Patient demographics including age, race, gender, and insurance status were gathered. The patient's age was assessed with respect to the date of the qualifying pancreatic biopsy.

Statistical analysis

When analyzing and comparing patient biopsy modalities with insurance status, a chi-square analysis was done in order to understand which variables should be included in the multivariable binary logistic analysis with a 95% confidence interval (CI). Additionally, another multivariable binary logistic regression was used to perform an analysis to examine the patient population's demographic and social variables. Statistical significance was determined with a cut-off of a level of p <0.001. When analyzing the average LOS amongst different biopsy methods, a one-way analysis of variance (ANOVA) test was utilized in order to compare the averages for LOS with regard to different biopsy methods, with a significance of p <0.001. When analyzing hospital stay complications in patients undergoing different biopsy methods, a chi-square analysis was performed for all categorical variables with a 95%CI. Using a multivariable logistic regression medical, complications (MI, pancreatitis, pneumonia, UTI, and ARF) and demographic variables were compared with a significance level of p <0.001.

## Results

We identified 824,162 patients with pancreatic cancer from 2001-2013 in the NIS database. Of these, 50,194 underwent PB, 3,817 underwent EB, 11,668 underwent SB, and 1,102 underwent a combination of the three biopsy modalities. Out of all the patients who underwent PB, the OR of a patient having Medicaid was 1.35, Medicare was 1.19, and an uninsured status was 2.00. For those patients who underwent EB, the OR of having Medicaid was 1.17, Medicare was 1.16, and uninsured status was 1.45. For those patients who underwent SB, the OR of having Medicaid was 0.79, Medicare was 1.04, and an uninsured status was 1.04 (Table 1).

**Table 1:** Predictors of Biopsy Method based on Insurance Status in the Study Population *Significance level p<0.001

Insurance cohort	P-value	Odds ratio (95% CI)
Private Insurance	Reference	
Medicaid		
Surgical biopsy	0.000^*^	0.79 (0.72-0.87)
Percutaneous biopsy	0.000^*^	1.35 (1.29-1.41)
Endoscopic biopsy	0.064	1.17 (0.99-1.37)
Medicare		
Surgical biopsy	0.182	1.04 (0.98-1.10)
Percutaneous biopsy	0.000^*^	1.19 (1.15-1.22)
Endoscopic biopsy	0.005	1.16 (1.05-1.28)
Uninsured		
Surgical biopsy	0.584	1.04 (0.91-1.18)
Percutaneous biopsy	0.000^*^	2.00 (1.90-2.11)
Endoscopic biopsy	0.000^*^	1.45 (1.18-1.79)

When looking at the mean LOS, patients who underwent PB had a mean LOS of 9.31 days with a standard deviation (SD) of 7.92 days. Those who underwent EB had a mean LOS of 8.61 days with an SD of 8.26 days. Those who underwent SB had a mean LOS of 12.82 days with an SD of 10.36. Finally, those who underwent a combination of the biopsies had a mean LOS of 14.77 days with an SD of 9.63 days. The mean LOS amongst all patients undergoing biopsy was 9.87 days with an SD of 8.57 days (Table 2).

**Table 2:** Average LOS for Endoscopic, Percutaneous, and Surgical Biopsy Patients *Significance level p<0.001 LOS: length of hospital stay

Variable	Mean (95% CI)	Standard deviation (Standard error)
Endoscopic biopsy	8.61 (8.34-8.89)	8.26 (0.14)
Percutaneous biopsy	9.31 (9.24-9.38)	8.00 (0.04)
Surgical biopsy	12.82 (12.62-13.01)	10.35 (0.10)
Combination	14.77 (14.17-15.36)	9.63 (0.30)
Total	9.87 (9.80-9.94)	8.57 (0.03)

When looking at patients who developed a MI as a complication of biopsy (Table 3), those who underwent SB had the highest OR compared to PB (1.49 vs. 0.66 respectively). Additionally, patients with pancreatic cancer with an age of 80 and above, Asian/Pacific Islander/Native American, and those with Medicare had the highest OR amongst their groups (4.33, 1.10, and 1.20, respectively). 

**Table 3:** Predictors of Myocardial Infarction (MI) in the Study Population *Significance level p<0.001

Variable	P-value	Odds ratio (95% CI)
Age (years)		
19 to 20	Reference category	
30 to 50	0.395	1.48 (0.69-3.64)
51 to 60	0.098	2.13 (0.87-5.23)
61 to 79	0.003	3.95 (1.61-9.67)
≥ 80	0.000^*^	4.33 (1.77-10.62)
Race		
Caucasian	Reference category	
African American	0.000^*^	0.81 (0.76-0.87)
Hispanic	0.305	0.96 (0.88-1.04)
Asian, Pacific Islander, Native American	0.030	1.10 (1.01-1.20)
Gender		
Males	Reference category	
Females	0.000^*^	0.79 (0.76-0.82)
Insurance status		
Private insurance	Reference category	
Medicaid	0.003	0.84 (0.75-0.94)
Medicare	0.000^*^	1.20 (1.14-1.27)
No insurance	0.466	0.94 (0.81-1.10)
Other insurance status	0.005	0.79 (0.67-0.93)
Biopsy Type		
No percutaneous biopsy	Reference category	
Percutaneous biopsy	0.000^*^	0.66 (0.59-0.72)
Biopsy Type		
No endoscopic biopsy	Reference category	
Endoscopic biopsy	0.081	0.74 (0.53-1.04)
Biopsy Type		
No surgical biopsy	Reference category	
Surgical biopsy	0.000^*^	1.49 (1.29-1.72)

When looking at patients who developed acute renal failure (ARF) as a complication (Table 4), those who underwent EB had a higher OR compared to those who underwent SB (1.41 vs. 0.80 respectively). Additionally, those with an age of 80 and above, African Americans, and those with Medicaid or Medicare had the highest OR amongst their groups (3.54, 1.74, and 1.23 respectively).

**Table 4:** Predictors of Acute Renal Failure (ARF) in the Study Population *Significance level p<0.001

Variable	P-value	Odds ratio (95% CI)
Age (years)		
19 to 20	Reference category	
30 to 50	0.000^*^	1.76 (1.28-2.43)
51 to 60	0.000^*^	2.28 (1.66-3.14)
61 to 79	0.000^*^	2.86 (2.08-3.93)
≥ 80	0.000^*^	3.54 (2.57-4.87)
Race		
Caucasian	Reference category	
African American	0.000^*^	1.74 (1.70-1.79)
Hispanic	0.000^*^	1.14 (1.09-1.18)
Asian, Pacific Islander, Native American	0.000^*^	1.20 (1.15-1.25)
Gender		
Males	Reference category	
Females	0.000^*^	0.73 (0.72-0.74)
Insurance status		
Private insurance	Reference category	
Medicaid	0.000^*^	1.23 (1.18-1.28)
Medicare	0.000^*^	1.23 (1.20-1.26)
No insurance	0.000^*^	1.20 (1.13-1.28)
Other insurance status	0.000^*^	0.87 (0.81-0.94)
Biopsy type		
No percutaneous	Reference category	
Percutaneous	0.005	1.06 (1.02-1.10)
Biopsy type		
No endoscopic	Reference category	
Endoscopic	0.000^*^	1.41 (1.25-1.58)
Biopsy type		
No surgical	Reference category	
Surgical	0.000^*^	0.80 (0.73-0.87)

When looking at patients who developed a UTI as a complication (Table 5), those who underwent EB had the highest OR, while those who underwent SB had the lowest OR (1.39 vs. 0.79 respectively). Additionally, those with an age of 80 and above, African Americans, females, and those with Medicaid had the highest OR amongst their groups to develop a UTI (2.34, 1.17, 2.38, and 1.39 respectively).

**Table 5:** Predictors of Urinary Tract Infection (UTI) in the Study Population *Significance level p<0.001

Variable	P-value	Odds ratio (95% CI)
Age (years)		
19 to 20	Reference category	
30 to 50	0.639	1.06 (0.84-1.34)
51 to 60	0.054	1.26 (1.00-1.59)
61 to 79	0.000^*^	1.64 (1.30-2.07)
≥ 80	.000^*^	2.34 (1.85-2.95)
Race		
Caucasian	Reference category	
African American	0.000^*^	1.17 (1.14-1.20)
Hispanic	0.000^*^	1.13 (1.09-1.17)
Asian, Pacific Islander, Native American	0.002	1.07 (1.02-1.11)
Gender		
Males	Reference category	
Females	0.000^*^	2.38 (2.33-2.43)
Insurance status		
Private insurance	Reference category	
Medicaid	0.000^*^	1.39 (1.33-1.44)
Medicare	0.000^*^	1.36 (1.32-1.40)
No insurance	0.000^*^	1.20 (1.13-1.28)
Other insurance status	0.637	
Biopsy type		
No percutaneous	Reference category	
Percutaneous	0.000^*^	1.11 (1.07-1.15)
Biopsy type		
No endoscopic	Reference category	
Endoscopic	0.000^*^	1.39 (1.24-1.55)
Biopsy type		
No surgical	Reference category	
Surgical	0.000^*^	0.79 (0.72-0.86)

When looking at patients who develop pneumonia as a complication (Table 6), all biopsy types were less likely to have pneumonia during their hospital stay. Those who underwent EB had the lowest OR at 0.55. Additionally, Asian/Pacific Islander/Native Americans and those with Medicare had the highest OR amongst their groups (1.11 and 1.22 respectively).

**Table 6:** Predictors of Pneumonia (PNA) in the Study Population *Significance level p<0.001

Variable	P-value	Odds ratio (95% CI)
Age (years)		
19 to 20	Reference category	
30 to 50	0.070	0.79 (0.61-1.02)
51 to 60	0.694	0.95 (0.74-1.23)
61 to 79	0.425	1.11 (0.86-1.43)
≥ 80	0.135	1.22 (0.94-1.57)
Race		
Caucasian	Reference category	
African American	0.517	0.99 (0.95-1.02)
Hispanic	0.528	1.01 (0.97-1.06)
Asian, Pacific Islander, Native American	0.000^*^	1.11 (1.05-1.16)
Gender		
Males	Reference category	
Females	0.004	0.80 (0.77-0.81)
Insurance status		
Private insurance	Reference category	
Medicaid	0.000^*^	1.17 (1.11-1.23)
Medicare	0.000^*^	1.22 (1.18-1.26)
No insurance	0.902	1.00 (0.92-1.08)
Other insurance status	0.697	1.02 (0.94-1.10)
Biopsy type		
No percutaneous	Reference category	
Percutaneous	0.000^*^	0.59 (0.56-0.62)
Biopsy type		
No endoscopic	Reference category	
Endoscopic	0.000^*^	0.55 (0.44-0.67)
Biopsy type		
No surgical	Reference category	
Surgical	0.000^*^	0.84 (0.76-0.93)

When looking at patients who develop pancreatitis as a complication (Table 7), those who underwent EB had the highest OR compared to percutaneous and surgical methods (4.13 vs. 3.03 vs. 1.99 respectively). Additionally, African Americans and Asian/Pacific Islander/Native Americans had the same OR at 1.1, while those with no insurance had the highest OR at 1.33.

**Table 7:** Predictors of Pancreatitis in the Study Population *Significance level p<0.001

Variable	P-value	Odds ratio (95% CI)
Age (Years)		
19 to 20	Reference category	
30 to 50	0.269	0.88 (0.70-1.11)
51 to 60	0.006	0.72 (0.57-0.91)
61 to 79	0.000^*^	0.56 (0.45-0.71)
≥ 80	0.000^*^	0.59 (0.46-0.74)
Race		
Caucasian	Reference category	
African American	0.000^*^	1.13 (1.09-1.18)
Hispanic	0.926	1.00 (0.95-1.05)
Asian, Pacific Islander, Native American	0.000^*^	1.13 (1.07-1.20)
Gender		
Males	Reference category	
Females	0.004	0.96 (0.94-0.99)
Insurance status		
Private insurance	Reference category	
Medicaid	0.000^*^	1.14 (1.09-1.21)
Medicare	0.075	1.03 (1.00-1.07)
No insurance	0.000^*^	1.33 (1.24-1.43)
Other insurance status	0.000^*^	0.77 (0.70-0.85)
Biopsy type		
No percutaneous	Reference category	
Percutaneous	0.000^*^	3.03 (2.92-3.14)
Biopsy type		
No endoscopic	Reference category	
Endoscopic	0.000^*^	4.13 (3.71-4.59)
Biopsy type		
No surgical	Reference category	
Surgical	0.000^*^	1.99 (1.82-2.16)

## Discussion

Pancreatic cancer continues to be one of the major contributors to cancer-related deaths worldwide with only 10-20% survival over five years [1]. Though tremendous advances have been made in the treatment of other malignancies, progress in pancreatic cancer has been relatively slow while its relative disease burden continues to increase. In the absence of these developments, progress can be made in investigating disparities in the distribution of healthcare and the complications within the disease workup.

With regard to insurance status, our results demonstrated that both uninsured and Medicaid patients were more likely to undergo PB, and less likely to undergo SB, despite a lack of guidelines as to how to choose between them. This may represent a discrepancy in access to health care. While there are no studies looking at the impact of insurance status on biopsy methods, it has been well documented that patients with pancreatic cancer, like other malignancies, experience disparities in stage at presentation and treatment based on race, ethnicity, insurance status, marital status and geographic location [11-16]. Furthermore, these differences cannot be explained by discrepancies in screening as there are no approved screening options for pancreatic cancer [17,18].

One hypothesis for our findings is that an SB is more costly and time-consuming compared to a PB. Patients in areas of middle to high insurance coverage have been shown to be more likely to undergo surgery compared to more underserved areas, and thus would probably be more likely to undergo SB. The discrepancies seen in staging at the time of presentation based on social determinants of health, such as race and insurance status, may also preclude a surgical resection, and thus an SB. While endoscopic methods may provide a third option, this is limited to select locations that have specialized equipment and advanced endoscopists, making it difficult for many patients to access [19].

The second question that this study looked at was if LOS differed between biopsy types. We found that patients who underwent EB had the shortest LOS by at least one day compared to those who underwent PB, SB, or a combination of the three biopsies. Additionally, patients who underwent SB stayed an average of three more days and those who underwent a combination of biopsies had the greatest LOS. In recent years, the average hospital day is associated with a cost of upwards of $4,000 [20,21]. Therefore, appropriate determination for biopsy type would contribute to significant healthcare utilization budget reduction.

Though no previous studies have compared EB- and PB-related LOS, there have been several studies comparing endoscopic and percutaneous drainage of pancreatic fluid collections, which showed endoscopic draining being associated with lower rates of reintervention, adverse events, and shorter hospital stays [15,22-24]. EBs are not only favored because of this but also because there is a theoretical risk of peritoneal implantation of the tumor with PB, which may occur along the needle tract. However, EB may not be appropriate for lesions at the tail of the pancreas [25].

Although similar diagnostic accuracy can be achieved, SBs are generally used as a last resort as they are both time-consuming and associated with an increased risk [26-28]. Patients who undergo SB tend to have a higher tumor burden and advancement of the disease, which is reflected in the LOS. Finally, we expect those who undergo a combination of biopsy methods to have the highest LOS likely due to one biopsy method producing inconclusive results, or multiple pancreatic lesions requiring different modalities, which will take additional time to schedule and complete.

Based on this data alone, we recommend most eligible patients undergo EB to not only reduce hospital stay and hospital burden but also reduce complications that may occur during a prolonged hospital stay. Future studies comparing the readmission rate following EB, PB, and SB may further strengthen this position.

The final question we investigated was if different biopsy methods had an increased risk of complications (MI, ARF, UTI, pneumonia, and pancreatitis) during the hospital stay. We found that patients who underwent EB were more likely to develop ARF, UTI, and pancreatitis compared to those who underwent SB. This may be due to the closed and operator-dependent nature of endoscopic ultrasound requiring advanced techniques to identify anatomy by ultrasound.

However, it is also important to note that these complications may also have an independent component from the biopsy method. Though our logistic regression demonstrated a significant (<0.001) relationship between method and complications, we also found that patients with an age of over 80 were more likely to develop an MI, UTI, and ARF during their hospital stay. Furthermore, we found that Asian/Pacific Islander/Native Americans were most likely to develop MI, pneumonia, and pancreatitis, while African Americans were most likely to develop ARF and UTI. Similarly, we found insurance status and sex to be associated with the likelihood of different complications. Demographic variables including race and socioeconomic status have been linked to disparities in care and complication risks. However, these systemic biases have also assisted in changing treatment guidelines allowing for improved care. Understanding the role of demographics in patients with pancreatic lesions and their access to biopsy methods will help assist diagnostic guidelines thereby improving patient morbidity and mortality [29-30].

Given the multiple and complex risk factors of hospital complications, it is difficult to conclude whether one method truly increases the risk of one complication versus the other. Factors like tumor burden, staging, body mass index (BMI), and a patient’s comorbidities likely also play a role and should be investigated in future studies. Additional limitations to our study include identifying the size and location of the pancreatic biopsy, as this may greatly impact the decision-making process of the biopsy modality. Likewise, income level may be a better predictor of biopsy type rather than insurance status, given the cost in time and money of surgical procedures.

## Conclusions

There are no current standardized guidelines or risk stratification scores being used to help determine whether patients should undergo PB, EB, or SB. Currently, this decision is made by the physician based on tumor size and location. Our study shows insurance status plays a role in which modality patients undergo. Furthermore, our data suggest that EBs result in the shortest LOS, while SB and combination biopsies result in the longest LOS. Finally, our study showed that different biopsy methods do in fact change the risk of developing different hospital complications. However, this might be confounded by other variables not measured. Future randomized controlled studies can be completed to control for tumor size, location, and a patient’s comorbidities.
